# Bidirectional Estrogen-Like Effects of Genistein on Murine Experimental Autoimmune Ovarian Disease

**DOI:** 10.3390/ijms17111855

**Published:** 2016-11-08

**Authors:** Qiao Ding, Yuxiao Wang, Na Li, Kexue Zhu, Jielun Hu, Sunan Wang, Fan Zhu, Shaoping Nie

**Affiliations:** 1 State Key Laboratory of Food Science and Technology, Nanchang University, Nanchang 330047, China; ncuskdingqiao@163.com (Q.D.); 18641603755@163.com (Y.W.); nali20088888@126.com (N.L.); Hujielun@ncu.edu.cn (J.H.); 2 Spice and Beverage Research Institute, Chinese Academy of Tropical Agricultural Sciences, Wanning 571533, China; zhukexue163@163.com; 3 Canadian Food and Wine Institute, Niagara College, 135 Taylor Road, Niagara-on-the-Lake, ON L0S 1J0, Canada; swang8000@gmail.com; 4 School of Chemical Sciences, University of Auckland, Private Bag 92019, New Zealand; fzhu5@yahoo.com

**Keywords:** autoimmune ovarian disease, genistein, zona pellucida, oophoritis, estradiol

## Abstract

This study was to investigate the bidirectional estrogen-like effects of genistein on murine experimental autoimmune ovarian disease (AOD). Female BALB/c mice were induced by immunization with a peptide from murine zona pellucida. The changes of estrous cycle, ovarian histomorphology were measured, and the levels of serum sex hormone were analyzed using radioimmunoassay. Proliferative responses of the ovary were also determined by immunohistochemistry. Administration of 25 or 45 mg/kg body weight genistein enhanced ovary development with changes in serum sex hormone levels and proliferative responses. Meanwhile, the proportions of growing and mature follicles increased and the incidence of autoimmune oophoritis decreased, which exhibited normal ovarian morphology in administration of 25 or 45 mg/kg body weight genistein, while a lower dose (5 mg/kg body weight genistein) produced the opposite effect. These findings suggest that genistein exerts bidirectional estrogen-like effects on murine experimental AOD, while a high dose (45 mg/kg body weight) of genistein may suppress AOD.

## 1. Introduction

Premature ovarian failure (POF), also named premature ovarian insufficiency, results in the dysfunction of the ovaries and consequent insufficiency of estrogen in women under the age of 40 [1]. Patients with POF suffer from anovulation, infertility and menopausal symptoms. POF has been reported to play a causative role in many diseases, including osteoporosis, hypothyroidism, Addison’s disease, and other auto-immune disorders. Statistically, the risk of POF for women under the age of 40 is 1% and under the age of 30 is 0.1% [2,3,4]. Multicausal pathogenesis had been suggested in the development of POF, such as genetic abnormalities, previous ovarian surgery, systemic chemotherapy and radiotherapy, infections, enzymatic factors and autoimmune disease [5].

Autoimmune ovarian disease (AOD) is a chronic inflammatory disease. AOD was closely related with lymphocytic infiltration of ovarian follicles in females with POF, which can be induced by susceptible strains of animals via immunizing with zone pellucid (ZP) antigens and adjuvant [6,7,8]. The mammalian ZP is extracellular glycoprotein surrounding the oocytes and plays an important role in spermatozoa–oocyte interaction and fertilization. Furthermore, ZP has been certified to affect the development of follicles. Many researches showed that the presence of antibodies in ZP antigens could alter ovarian function and histology by interfering with cellular immune response [9,10,11].

There are many investigations that have evaluated the functions of phyto-oestrogens, including isoflavones, coumestans and lignans. Soy isoflavones, particularly daidzein and genistein, are beneficial for breast cancer, neuronal injury, prostate cancer, sexual dysfunction, osteoporosis and menopausal symptoms [12,13]. The potential therapeutic benefits are derived from the estrogenic effect of soy isoflavones [14,15,16]. Therefore, soy isoflavones could be used as safe, natural alternatives to traditional hormonal therapies [17,18]. However, experimental evidences are controversial when the safety and efficacy of dietary supplements containing soy isoflavones was regarded [19,20,21,22]. Recently, a review summarized that the beneficial and harmful effects of phyto-estrogens may be related to the exposure time, dosage and form in animals [13]. Genistein could stimulate the proliferation of ERα-positive cells including MCF-7 and T47D cells, but did not stimulate the proliferation of ERα-negative cells including MDA-MB-435 cells. While, another report gave the opposite result, that genistein had an antiproliferative effect in MCF-7 breast cancer cells [23]. Seo et al. concluded that the utilization of phytoestrogens in postmenopausal women might be detrimental [24].Thus, it appears that more researches should be done to clarify the role of soy-based products in the reduction of menopausal symptoms.

Genistein has been reported to bind to and signal through estrogen receptors, thereby making the major site of estrogen receptors-ovary as the target tissue for genistein [25]. Therefore, in order to investigate the role of genistein in experimental AOD, genistein was orally administered to mouse during the acute stage of autoimmune oophoritis. Diverse techniques were employed to examine the changes of estrogen-dependent target tissue and determine ovary and estrogenic levels in the mouse serum.

## 2. Results

### 2.1. Regularization of the Estrous Cycle by Oral Administration of Genistein

The estrous cycles of normal female mice were regular and lasted 4–6 days: proestrus and metestrus for 1 day, estrus and diestrus for 1–2 days. Irregular cycles occurred before amenorrhea happened which is the character of POF. The patterns of estrous cycles were categorized based on an increasing degree of abnormality from I to IV, as per the standard in the report given by Bagavant et al. [26].

**Figure 1:**
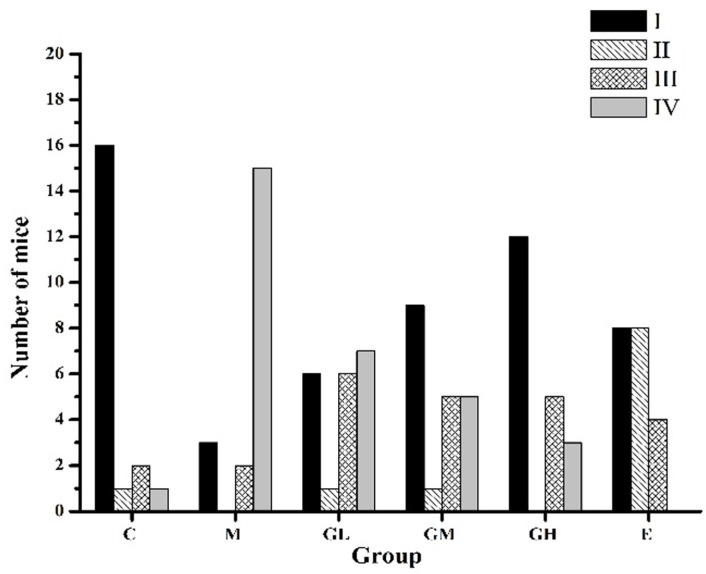
Effects of genistein on estrous cycle patterns of BALB/c female mice. Four patterns of abnormal estrous cycles were graded in an increasing order of abnormality (I–IV) as follows: I: normal; II: regular cycles with a shortened estrus; III: irregular cycles with a prolonged diestrus and normal or prolonged estrus; IV: no cyclicity. C: control group; M: model group; GL: low-dose of genistein (5 mg/kg body weight) therapeutic group; GM: moderate-dose of genistein (25 mg/kg body weight) therapeutic group; GH: high-dose of genistein (45 mg/kg body weight) therapeutic group; E: estradiol (1 mg/kg body weight) therapeutic group.

As shown in Figure 1, estrous cycle patterns changed during genistein administration. Normal estrous cycles (category I) were 85% of the mice in control group (C); however, only 15% of mice in the model group (M) were in normal cycles. The numbers of mice in normal cycles in C and M groups were significantly different (*p* < 0.01). In contrast, the estrous cycles of mice in genistein and estradiol groups were more regular. It was observed that 30% (low-dose of genistein (5 mg/kg body weight) therapeutic group (GL group)), 45% (moderate-dose of genistein (25 mg/kg body weight) therapeutic group (GM group)), 60% (high-dose of genistein (45 mg/kg body weight) therapeutic group (GH group)), 40% (estradiol (1 mg/kg body weight) therapeutic group (E group)) of mice cycled normally, respectively. The numbers of mice in normal estrous cycles in the GH group were significantly higher than those in the M group (*p* < 0.05). Furthermore, fewer mice in the GH groups showed no cycles (category IV) (15%) than those in the M group (75%, *p* < 0.01). The E group tended also to have more mice with regular cycles (40%) and shortened estrus (40%) compared to the C group (5%).

### 2.2. Estradiol Increased While Those that Are Follicle-Stimulating Hormone and Luteinizing Hormone Decreased after Oral Administration of Genistein

For women with amenorrhea, it would be reasonable to measure basal follicle-stimulating hormone (FSH) and estradiol (E_2_) concentrations on at least two occasions if the value of FSH is at all elevated [27]. In addition, luteinizing hormone (LH) and FSH value >30 IU/L, and levels of E_2_ < 50 pg/mL are typical for women with absent or nonfunctioning follicles.

As shown in Table 1, the levels of FSH and LH increased significantly, but E_2_ decreased significantly in the M group compared to the C group (*p* < 0.05) in the 1st, 20th, 30th, and 50th day. After administration of genistein, the levels of FSH and LH decreased and E_2_ enhanced to different extents on the 1st, 20th, 30th, and 50th day. For instance, the levels of FSH reduced by 3%, 23% and 28% in GL, GM and GH groups compared to the M group on the 50th day, respectively. The decrease in GM and GH groups was significantly different (*p* < 0.05), while in GL group there was no significant difference. Also, the changes of LH were similar to the FSH. However, the levels of E_2_ increased by 37%, 37% and 46% in GL, GM and GH groups compared to the M group on the 30th day, respectively. The enhancement of E_2_ in the GH group was significantly different (*p* < 0.05). In addition, the levels of prolactin (PRL) increased and the levels of testosterone (T) decreased in administration of genistein groups compared to M group with no significant difference (*p* > 0.05). Meanwhile, FSH and LH reduced and E_2_ enhanced in the E group during administration of estradiol.

### 2.3. Decreased Morbidity of Oophoritis after Oral Administration of Genistein

**Figure 2:**
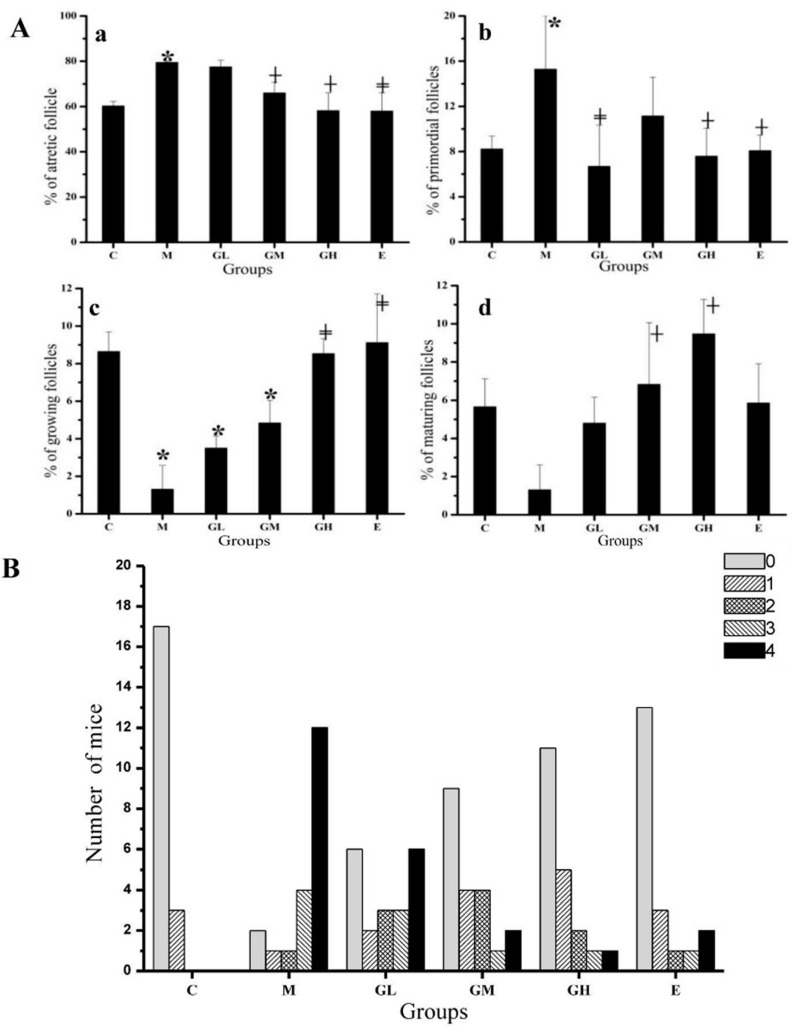
The follicles ratio of every stage and oophoritis morbidity in the ovary. (**A**) The follicles ratio of every stage (**a**: atretic follicles; **b**: primordial follicles; **c**: growing follicles; **d**: mature follicles); (**B**) Autoimmune oophoritis was classified according to increasing severity from 1 to 4, 0: normal with no oophoritis. * *p* < 0.05 compared to control group; ^┼^ *p* < 0.05 compared to the model group; ^╪^ *p* < 0.01 compared to the model group.

When mice were immunized with ZP3, inflammation developed in the ovarian and in the growing and mature follicles. Moreover, some ovaries exhibited a significant loss of growing and mature follicles. As shown in Figure 2A, the ratio of growing follicles reduced significantly in the M group on day 78 compared to the C group (Figure 2c). The numbers of atretic and primordial follicles in the M group were increased (*p* < 0.05) relative to mice in the C group on day 78 (Figure 2a,b). The loss of follicles was concomitant with a decrease in corpora lutea [28], indicating that ovarian function was disrupted. Following exposure to genistein and estradiol, the ratio of atretic and primordial follicles decreased, and growing and mature follicles increased compared to the M group, respectively. Moreover, the changes in follicles ratio of every stage in the GM and GH groups were significantly different compared with the M group (*p* < 0.05). While there was no difference in the ratios of atretic, growing and mature follicles between the M group and GL group (*p* > 0.05). However, the changes of follicles showed no difference between mice administration of genistein and estradiol (*p* > 0.05).

The oophoritis on an increasing severity from 1 to 4 was shown in Figure 2B. The oophoritis morbidity was 15% in the C group; however, 90% of the mice in the M group had oophoritis. The oophoritis morbidity in the GL, GM and GH groups was 70%, 55% and 45%, respectively. The oophoritis morbidity decreased significantly in the GL, GM and GH groups compared to the M group (*p* < 0.01). While the oophoritis morbidity in the E group was 35%, which declined significantly compared to the M group (*p* < 0.01). However, the mice with oophoritis in the GM and GH groups were focused on the level 1 and 2, while for mice in the GL group, they were mainly on level 4, which is similar to the M group.

### 2.4. Proliferative Responses of the Ovary

**Figure 3:**
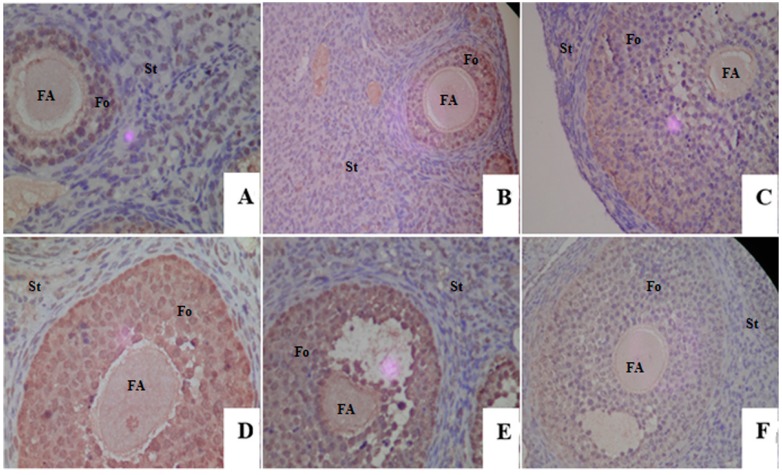
Photomicrograph of PCNA from growing follicles in each group. (**A**) Control group; (**B**) model group; (**C**) estradiol group; (**D**) low-dose genistein group; (**E**) moderate-dose genistein group; (**F**) high-dose genistein group. The expression of PCNA-positive was brown in color, while blue in color for PCNA-negative expression. Original magnification 400×. Fo: follicle; St: stroma; FA: follicle antrum.

The proliferation rate of the ovary follicles was determined by using an immunohistochemical staining of proliferating cell nuclear antigen (PCNA) protein (Figure 3), which was an accessory protein to δ polymerase, and was closely related to DNA replication, DNA repair and cell cycle progression [29]. Thus, PCNA was considered as a marker for the ovarian follicle growth. For all groups, the expression of PCNA-positive (brown in color) was visible not only in the follicles, but also in the stroma. Comparing to the C group, the strength and density of PCNA expression were much higher and mainly on the follicles in the M group. For mice exposure to genistein and estradiol, the expression of PCNA was focused on the follicles and changed differently in ovaries of mice in different groups. As shown in Figure 3, the strength and density of PCNA-positive expression in the E_2_ and GM groups were similar to the M group, while for the GL group the strength and density of PCNA-positive were significantly higher than that in the M group. However, the expression of PCNA in the GH group was similar to the C group and significantly lower compared to the M group.

## 3. Discussion

Autoimmune ovarian disease is a known cause of human premature ovarian failure. AOD induction by immunizing with ZP3, an ovary-specific glycoprotein, is simple and rapid. Also, the experimental murine model was developed in 1991 [30] and had been successfully exploited in investigating some fundamental issues of self-tolerance and autoimmune disease mechanisms [31]. The irregular estrous cycles, changes in endocrine hormone levels and high oophoritis morbidity in the M group indicated that the autoimmune ovarian disease model was successful. The influence of ZP3 on the ovarian lifespan might be a continuous process through the steady enhancement of FSH and LH and decline of E_2_. Therefore, the AOD model was widely used to investigate the therapeutic efficacy of materials for POF.

In this study, the mice received the diet supplemented with genistein at different doses (5, 25, 45 mg/kg body weight). The estrous cycle was convenient and useful for monitoring the health state of female mice. The results showed that the estrous cycles were more regular after administration of genistein and eatradiol relative to the M group. Genistein could improve the occurrence of irregular cycles, which was consistent with Zhuang et al. [32]. The levels of endocrine hormone were reported to influence the development of follicles. FSH and LH had a synergistic effect on regulating follicular development and differentiation, E_2_ was related to secretion of follicles. Our results found that consumption of genistein enhanced the E_2_ level and could attenuate the preovulatory surge of LH and FSH in GM and GH groups compared to the M group. However, there was no significant difference in the levels of FSH, LH and E_2_ in GL group compared to the M group. Furthermore, the level of PRL showed a decreasing tendency, while for level of T was an opposite tendency with no significant difference when compared to the M group. These results were proved by multiple studies that had documented the estrogenic activity of genistein which has an indirect effect on the hypothalamic-pituitary-gonadal axis [33,34,35]. Zin et al. [36] found that FSH increased and LH decreased in rats with 10 mg/kg body weight of genistein, FSH and LH both reduced in rats with 100 mg/kg body weight of genistein. They concluded that post-weaning exposure to genistein could influence development of the reproductive system through regulating hormones. Otherwise, the increase of growing and mature follicles, decrease of antral and primordial follicles, and the decline of oophoritis morbidity after administration of moderate and high dose of genistein indicated that genistein could improve the inflammation response in ovary. Xiao et al. found that amniotic fluid stem cells could prevent follicle atresia and sustain the healthy follicles in the ovary of mice which were induced POF by chemotherapeutic drugs [37].

Genistein could have a proliferative or antiproliferative effect depending on the levels of endogenous estrogen, life stage and tumor types. The physiologies such as development and differentiation of ovary could be modified after dietary phytoestrogen exposure [38]. When genistein effects relate to proliferation, it was meaningful to determine the expression profiles of proliferation markers like PCNA [39]. In the ovary, the expression of PCNA may affect follicular growth by regulating PCNA-dependent granulose cell proliferation in follicles [40]. In the present study, PCNA was mainly expressed in the ovarian follicles. The expression of PCNA in the GH group was similar to the C group and significantly lower compared to the M group. However, the expression in the GL group was higher than the M group. Interestingly, the expressions of PCNA after exposure to genistein were consistent with the histomorphological results regarding the follicular development.

## 4. Materials and Methods

### 4.1. Animals and Chemicals

One hundred and twenty female mice BALB/c (7–9 weeks) were obtained from the HFK BIOSCIENCE Co., Ltd. (Beijing, China). Before starting the experiments, all the animals were housed at an ambient temperature of 25 ± 2 °C, 12/12 h of light-dark cycle with ad libitum food and water for 1 week. After acclimation, a total of 120 mice were randomly divided into six groups (*n* = 20 per group): C group, M group, GL group, GM group, GH group, and E group. All groups except for C group received experimental AOD via immunization. In addition, there were not any estrogenic compounds exposure in diet, caging and bedding during the period. Care and treatment of the animals were based on approved protocols in accordance with the NIH guidelines (NIH Publication 85-23, 1996).

The murine ZP3 peptide was synthesized by an automatic peptide synthesizer, and with a purity of more than 90% as determined by HPLC analysis. The amino acid composition was verified by amino acid analysis and the amino acid sequence of the murine ZP3^330–342^ peptides was NSSSSQFQIHGPR.

Genistein was purchased from Ci Yuan Biotechnology Co., Ltd. (Shanxi, China) with a purity of more than 98% as determined by HPLC analysis. Estradiol and rabbit anti-mouse PCNA, a primary antibody, were obtained from Sigma (St. Louis, MO, USA). The goat anti-rabbit IgG secondary antibody was obtained from Beijing Zhongshan Jinqiao Biotechnology Co., Ltd. (Beijing, China). Other chemicals were of analytical grade and purchased from Zhongshan Jinqiao Biotechnology Co., Ltd. (Beijing, China).

### 4.2. Estrous Cycle Staging

The estrous cycles were determined by examining vaginal smears. Then, the estrous cycles were staged by vaginal cytological appearance, mainly on the proportions of leukocytes, nucleated epithelial cells and cornified squamous epithelial cells. The vaginal smears were taken according to the published method by Zhuang et al. [32].

### 4.3. Immunization and Induction of Genistein

**Figure 4:**
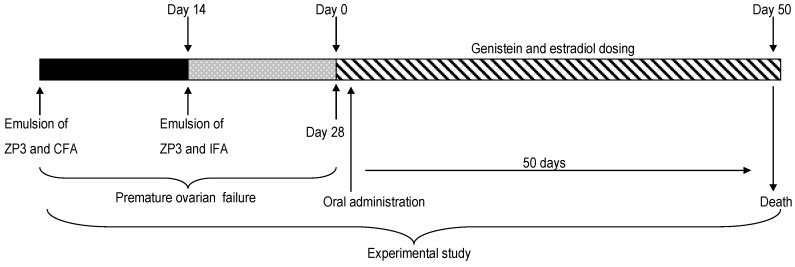
Schematic illustration of experimental design.

Ultrafiltered ZP3 peptides solution (1 mmol) was emulsified in an equal volume of complete Freund’s adjuvant (Mycobacterium tuberculosis, H37Ra strain; 0.16 mg/mouse). Mice were anesthetized by intraperitoneal injection (i.p.) of tribromoethanol. Each mouse except for C group was immunized subcutaneously in both hind footpads and received 0.15 mL of the mixture that contained 50 nmol of the peptides. After 14 days, 0.15 mL of the emulsion (1 mmol incomplete Freund’s adjuvant and ZP3) was injected into each mouse in the same position. C group received 0.15 mL of double-distilled water each time. Genistein and estradiol were administrated by daily gavage after the immunization (Figure 4: experimental study). Genistein was solubilized in DMSO to yield final concentrations of 0.75, 3.75 and 6.75 mg/mL. The doses of genistein in GL, GM, GH groups were 5, 25, 45 mg/kg body weight, respectively. Estradiol also was solubilized in DMSO solution (0.15 mg/mL (1 mg/kg body weight)) and was used as a positive control. Mice were given the same volume of DMSO in C group and M group. Mice were sacrificed on day 50 after treatment.

### 4.4. Detection of Serum Sex Hormone by Radio Immunity

Blood samples were collected from mice on day 1, 20, 30 and 50 after immunization and clotted at room temperature. Then, serum samples were obtained by centrifuging at 3287× *g* for 20 min, and frozen at −80 °C for analysis of sex hormones including E_2_, FSH, LH, PRL and T, using a solid-phase RIA kit (Diagnostic Products, Beijing Sino-uk institute of Biological Technology, Beijing, China). Aliquots of serum (300 μL or less) were assayed in sextuplicate.

### 4.5. Ovarian Histomorphology

At the end of the experimental period, animals were sacrificed. Ovaries were fixed in 10% formalin for 24 h and embedded in paraffin. Serial sections (5 μm) were stained with hematoxylin and eosin. Specimens were coded and examined by an independent observer who was blind to experimental details. Oophoritis was classified in an increasing severity from 1 to 4 based on a previous report [41].

### 4.6. Immunohistochemistry Analysis

For an immunohistochemical analysis of PCNA expression in the ovary, tissues were fixed in 10% formaldehyde solution and embedded in paraffin, blocked and cut into 5 μm sections. After rehydration, protein epitope retrieved with Tris-EDTA (10 mmol; pH 9) for 12–17 h at 60 °C. NOVA Detect Mouse Tissue Detection System protocol with specific primary antibodies against PCNA was used to detect the ovarian PCNA expression.

### 4.7. Statistical Analysis

Results were presented as mean ± S.D. Student’s *t*-test, rank-sum test, and one-way ANOVA were used for data analysis with SPASS 17.0 software (Version 17.0, Chicago, IL, USA). *p* < 0.05 was considered significant.

## 5. Conclusions

In conclusion, we demonstrated that orally administering moderate and high doses of genistein successfully suppressed experimental AOD in mice in this study, as evidenced by normalization of estrous cycle, decreasing levels of FSH and LH, increasing levels of E_2_ and alleviating abnormal ovarian histomorphology, especially at high doses. However, a significant improvement was not attained from low doses of genistein when compared to the M group. Comparing the serious side effects of traditional hormonal therapies for POF, there is an urgent need for natural alternatives. Our results proved that phytoestrogens such as genistein may be a promising, safe therapeutic agent for the treatment of premature ovarian failure. Therefore, it is reasonable to carry out further clinical research on genistein, its pharmacological dosage and weak effects for the treatment of POF.

## Figures and Tables

**Table 1:** Levels of sex hormone in mouse serum.

Time/Day	Group	E_2_/pg·mL^−1^	LH/mIU·mL^−1^	FSH/mIU·mL^−1^	PRL/ng·mL^−1^	T/ng·mL^−1^
1st	C	9.41 ± 0.45	30.15 ± 2.34	9.33 ± 1.01	9.28 ± 0.65	0.531 ± 0.062
	M	5.24 ± 0.90 ^a^	36.51 ± 0.51 ^aa^	9.90 ± 0.54	7.09 ± 0.65	0.544 ± 0.072
	GL	7.66 ± 0.36	31.41 ± 2.43 ^b^	8.68 ± 0.71	7.07 ± 1.02	0.448 ± 0.045
	GM	8.39 ± 0.22	31.65 ± 2.07 ^b^	7.15 ± 0.23	10.77 ± 0.20 ^b^	0.514 ± 0.055
	GH	9.17 ± 3.15 ^b^	34.23 ± 0.87	5.69 ± 1.71 ^aa,bb^	8.48 ± 0.36	0.492 ± 0.021
	E	6.45 ± 0.79	32.02 ± 1.03 ^b^	8.24 ± 0.52	8.74 ± 0.42	0.421 ± 0.018
20th	C	13.10 ± 8.57	31.50 ± 2.08 ^b^	7.60 ± 1.86 ^b^	9.01 ± 1.36	0.468 ± 0.006
	M	4.66 ± 0.56 ^aa^	36.33 ± 1.41	10.05 ± 1.11	7.45 ± 0.97	0.484 ± 0.020
	GL	9.09 ± 3.27	29.80 ± 1.94 ^bb,c^	7.57 ± 0.92	9.36 ± 1.39	0.463 ± 0.046
	GM	5.21 ± 0.85 ^aa^	36.39 ± 1.83 ^a^	8.96 ± 0.66	8.43 ± 1.08	0.432 ± 0.042
	GH	4.67 ± 0.45 ^aa^	32.56 ± 2.19 ^b^	6.84 ± 0.26 ^bb^	8.25 ± 0.67	0.479 ± 0.026
	E	4.79 ± 0.14 ^aa^	34.72 ± 3.98	8.71 ± 0.30	9.39 ± 0.74	0.457 ± 0.045
30th	C	4.32 ± 0.37	30.61 ± 0.37	8.36 ± 0.92	8.56 ± 1.23	0.397 ± 0.192
	M	3.01 ± 0.45 ^a^	36.87 ± 0.56 ^aa^	10.32 ± 0.12 ^a^	8.44 ± 0.75	0.437 ± 0.016
	GL	4.13 ± 0.22	32.35 ± 1.31 ^b^	8.89 ± 0.23	8.56 ± 0.52	0.414 ± 0.013
	GM	4.13 ± 0.81	36.73 ± 2.57 ^aa^	8.65 ± 0.42	7.23 ± 0.02 ^c^	0.449 ± 0.028
	GH	4.38 ± 0.57 ^b^	31.93 ± 0.90 ^b^	7.50 ± 0.19 ^b^	8.59 ± 0.39	0.691 ± 0.234 ^a,c^
	E	4.29 ± 0.16 ^b^	34.05 ± 0.61	8.48 ± 0.91	9.87 ± 1.20	0.399 ± 0.035
50th	C	4.42 ± 0.32	30.23 ± 1.62	9.02 ± 0.36	10.21 ± 1.31	0.470 ± 0.043
	M	3.03 ± 0.75 ^a^	34.65 ± 0.91 ^a^	11.35 ± 0.44 ^a^	7.52 ± 0.34 ^a,c^	0.486 ± 0.018
	GL	4.93 ± 0.78 ^b,c^	31.50 ± 1.96 ^b^	10.97 ± 0.93	8.76 ± 0.38	0.471 ± 0.010
	GM	4.54 ± 0.35 ^b^	30.15 ± 1.28 ^b,c^	8.78 ± 0.29 ^b^	7.78 ± 0.32 ^a,c^	0.461 ± 0.032
	GH	3.84 ± 0.24	34.68 ± 1.43 ^a^	8.14 ± 0.77 ^bb^	8.86 ± 0.56	0.469 ± 0.025
	E	3.51 ± 0.13	33.65 ± 1.16	9.34 ± 0.41 ^b^	9.98 ± 0.91	0.448 ± 0.019

All data are presented as mean ± S.D. ^a^: *p* < 0.05 compared to the control group; ^aa^: *p* < 0.01 compared to the control group; ^b^: *p* < 0.05 compared to the model group; ^bb^: *p* < 0.01 compared to the model group; ^c^: *p* < 0.05 compared to the estradiol group. C: control group; M: model group; GL: low-dose of genistein (5 mg/kg body weight) therapeutic group; GM: moderate-dose of genistein (25 mg/kg body weight) therapeutic group; GH: high-dose of genistein (45 mg/kg body weight) therapeutic group; E: estradiol (1 mg/kg body weight) therapeutic group.

## Abbreviations

AOD	autoimmune ovarian disease
POF	premature ovarian failure
ZP	zona pellucid
C group	control group
M group	model group
GL group	low-dose of genistein (5 mg/kg body weight) therapeutic group
GM group	moderate-dose of genistein (25 mg/kg body weight) therapeutic group
GH group	high-dose of genistein (45 mg/kg body weight) therapeutic group
E group	estradiol group
E_2_	estradiol
FSH	follicle-stimulating hormone
LH	luteinizing hormone
T	testosterone
PRL	prolactin
PCNA	proliferating cell nuclear antigen
